# A Plea for Higher Medical Education

**Published:** 1891-03

**Authors:** A. B. Patterson

**Affiliations:** Atlanta, Ga.


### A PLEA FOR HIGHER MEDICAL EDUCATION.

By A. B. PATTERSON, M. D., Atlanta, Ga.

"The Southern Doctors and their Work" is the subject of an editorial in the October number of THE ATLANTA MEDICAL AND SURGICAL JOURNAL. Medical education is a subject in which I have been deeply interested for a period of over twenty-three years, and I cannot let this opportune time pass without contributing a mite to the advancement of a cause in which the humblest citizen should feel a deep and personal interest. The editorial, to my mind, deserves more thought and consideration than I feel will be generally given it. The writer endeavors to encourage the doctors to observe and record their observations, and to help the "Southern profession." There is an air of complaint expressed in these lines, and, I think, very justly. The cause of the lethargy and backwardness in the profession South I do not believe is due to malarial influences operating on the nervous center, as a Washington doctor has so sarcastically suggested, nor is it due to so-called laziness. I am inclined to the view that it is largely due to our incomplete system of medical education and training, and the low standard that has existed in many of our institutions. It seems to me that these evils have had the effect of bringing into the medical profession, in many instances, an ignorant class of young men, who are wholly unprepared for the noble work before them ; who embark in the medical profession more to elude the plough handles than to elevate and advance the grand profession which is in need of the best talent of our country, devoted to thought and scientific investigation. Thus our ranks have become filled up with an inefficient class, whose sole aim is to eke out an easy existence. The effect has been to keep out the talented and ambitious young men, who fully appreciate the situation and engage in other pursuits. We are all conversant with the skepticism that pervades the mind of the unprofessional to a large class. Leaving out the doctor's individual popularity, one doctor is as good as another. This is an evidence of the lack of confidence in scientific medicine. This lack of confidence has made rich the pockets of the patent medicine men, and is a stumbling block to our advancement. I notice in the last few years there has been a greater demand among the unprofessional for better equipped doctors; they are beginning to recognize this progress in medicine as in other branches. The action of one college in adopting the three-course system, coupled with legislative acts creating a State board of examiners, and their thoroughness in eliminating from our ranks unqualified workers, I believe, has had much to do toward educating the public mind and in awakening a more lively interest in the minds of the profession.

The North is in advance of the South in scientific workers. The reason is found in the fact that their educational advantages are superior; they have equipped themselves with every facility for pursuing scientific investigations.

We have no advantages for acquainting ourselves with the ground work—the fundamental principles that underlie this grand structure of scientific medicine. Let every student who goes out to cope and battle with disease be a practical worker in histology, pathology, last but not least, bacteriology; conversant with the use of the microscope, the art of staining and mounting specimens, the cultivation and isolation of bacteria. Thus equipped the student of medicine becomes an intelligent observer and recorder, a logical thinker. Whether in the city or country he can press forward to the front, achieve a success that will make his name and work known over the civilized world. Atlanta is the city of the South. Let her educators put their heads together and establish and equip a laboratory that will be second to none in the United States, make a course in histology, pathology and bacteriology compulsatory in a regular course of medicine—give to our medical students all the advantages that can be derived by attending medical schools North. With such advantages and training our country, instead of being the refuge of ignorance and quackery, would become a light to the world. The "trash" found in our medical journals would give place to genuine scientific research; there will be more "eggs" and less "cackling" and less complaint.

I echo the plea as expressed in the *Southern Medical Record* of October. "A training school is a necessity. A trained nurse is a luxury." Then let Atlanta have a training school, and that, too, at once. It would be a waste of time to comment on the advantages of such an institution.

Every member of the profession will heartily approve the suggestion of the editor in saying there ought to be a chair of dietetics in every medical college.

27 *Old Capitol Building.*

